# Differential low uptake of free vitamin D supplements in preterm infants: the Quebec experience

**DOI:** 10.1186/s12887-014-0291-6

**Published:** 2014-11-30

**Authors:** Tarah Fatani, Atul K Sharma, Hope A Weiler, Odile Sheehy, Anick Bérard, Celia Rodd

**Affiliations:** Department of Pediatrics, Montreal Children’s Hospital, Montreal, Quebec Canada; Department of Pediatrics, Winnipeg Children’s Hospital, 685 William Avenue, FW302, Winnipeg, R3E 0Z2 Manitoba Canada; School of Dietetics and Human Nutrition, McGill University, Ste-Anne-de-Bellevue, Quebec, Canada; Research Center, Centre hospitalier universitaire Ste-Justine, Montreal, Quebec Canada; Faculty of Pharmacy, University of Montreal, Montreal, Quebec Canada

**Keywords:** Premature infants, Vitamin D, Supplementation, Mineralization, Fractures, Early preterm, Socioeconomic status, Education

## Abstract

**Background:**

Vitamin D is essential for bone mineralization, particularly in premature infants. For nearly 20 years, Quebec has offered a program of free vitamin D supplements via its public medication insurance plan *Régie de l’Assurance Maladie du Québec* (RAMQ). The objective of this study is to evaluate the number of preterm infants that obtained at least one bottle (50 doses) of vitamin D supplement through this program and to determine if uptake varied by gestational age.

**Methods:**

This was a retrospective cohort study of preterm infants covered by RAMQ and born from 1998 to 2008; all infants had 1 year of follow-up data regarding supplement use. Data were extracted from the Quebec Pregnancy Cohort, a linked administrative database and were stratified by early (<34 weeks) or late gestational age premature infants. The number of infants obtaining supplements was the primary outcome and their characteristics were compared across gestational age groups. Predictors for participation (obtaining at least 1 bottle) or adherence (2 or more bottles) were identified via logistic regression (GEE).

**Results:**

10288 infants were eligible; the percentage exposed to vitamin D was 24.5% (37.4%- early; 20.7%-late preterm infants, p < 0.001). The median number of bottles obtained was 2 for early and 1 for late preterms. For all premature infants, there was an apparent geometric decline in the infants obtaining subsequent bottles of supplements over the 12 month period. Additionally, there was a significant decline in program participation over time (OR = 0.90/year, 95% CI: 0.89-0.90) regardless of gestational age. Older or more educated mothers were positive predictors for participation. A prescription from a pediatrician significantly increased the odds of obtaining the supplement.

**Conclusion:**

Early preterm infants were more likely to obtain the supplement post-discharge; uptake was low and decreased with time for both age categories. Specifically, targeting late preterm infants and young mothers with less education could improve vitamin D uptake.

## Background

Preterm infants are at risk for bone health issues; the third trimester is a period of enhanced mineral transfer from mother and rapid bone mass accrual [1,2]. Since postnatal nutritional support cannot replicate the rich *in utero* environment, preterm infants may have fragility fractures before and after discharge; fracture rates range from 2-10% [1,2]. Measures to reduce such complications include special formulas or breast milk fortified with minerals, vitamins and protein [1,2]. Consensus has not yet been achieved on the dosage of vitamin D that preterm infants should receive but all preterm infants should receive a supplement as per established guidelines. European recommendations of 800–1000 IU/day are about double North American recommendations of 400 IU/d [3,4]. Many infants, particularly young preterm infants, are not capable of consuming sufficient formula to meet these recommendations, and breast fed infants also require supplementation because of the low concentration of vitamin D in breast milk [5]. Hence, there is a need for vitamin D supplementation for all preterm infants regardless of feeding modality.

**Figure 1:**
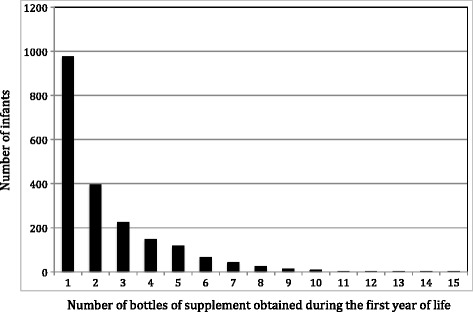
**Number of bottles of vitamin D supplements obtained over infancy in all preterm infants.**

Different jurisdictions promote vitamin D supplementation with various strategies; nevertheless, success has been variable and barriers still exist. In the UK, a system of coupons exchangeable for bottles of vitamin D has been instituted by the National Health Service (NHS) and has partially succeeded [6]. Turkey provides vitamin D via prescription [7]. We have recently reported that the province of Quebec has for 20 years utilized a program of free supplements via prescription without a major educational component [8]. Participation (obtaining 1 or more bottles) was limited to only 18% of full term infants during the period 1998–2009. Vitamin D exposure to infants declined over time in the face of a doubling of exclusive breastfeeding for the first 6 mo. of age from 2003-2009 [9]. This appeared to translate into a modest increase in the number of cases of rickets over this time [8].

The objective of this study is to evaluate the number of preterm infants that obtained at least one bottle (50 doses) of vitamin D supplement through this program and to determine if uptake varied by gestational age., using a linked administrative database (Quebec Pregnancy Cohort or QPC) [10]. We anticipated that the participation would be low, similar to term infants. Secondary objectives including assessing whether uptake by early preterm infants (born <34 weeks) might be different than infants born closer to term (late preterm 34- 366/7 weeks) [11]. Long term adherence (obtaining 2 or more bottles) was also examined, as were likely predictors, such as maternal education and physician specialty providing the prescription, which we have previously identified as important and modifiable factors in term infants in Quebec [8].

## Methods

### Study population

This was a retrospective analysis of an annual birth cohort of all preterm infants in Quebec born between January 1, 1998 and December 31, 2008. All data are obtained from the QPC, a validated, comprehensive and continuing population-based cohort [10,12,13]. This includes all pregnancies of women covered by the *Régie de l’Assurance Maladie du Québec* (RAMQ) medication insurance plan; about 30% of all pregnant women are covered by RAMQ while the rest have private group insurance. The QPC is created from a linkage of 3 administrative data bases including RAMQ (medical services and prescription claims), MED-ECHO (hospitalizations) and *Institut de la Statistique du Québec* (birth registry) using the maternal and child provincial health record numbers, date of births and names [10]. As described in more detail in the manuscript concerning term uptake, information such as prescription medications, socioeconomic status (SES), health care provider’s details, hospitalizations, and parental demographics are available in the QPC [8].

In 1994, the first vitamin D preparation was placed on the RAMQ formulary; this was free for social assistance recipients. Since 1997, supplements have been free via prescription for all participants of the RAMQ plan, with comparable coverage provided by all private group insurance programs.

Patient eligibility included all preterm infants (born <37 weeks) during the study period; these infants were further subdivided into early (<34 weeks) and late preterm infants (34 to 366/7 weeks) [11]. Their mothers needed to be covered by the RAMQ plan for the first 12 months post-delivery, and information about the duration of hospitalization was required. Infants with rare rachitic disorders such as renal failure, X-linked hypophosphatemic rickets, and infants and mothers treated with calcitriol were excluded using ICD-9 and ICD-10 codes.

### Data analysis

Our primary outcome was the number of preterm infants participating in this free vitamin D program in each calendar year between 1998 and 2009 (born 1998 till 2008 with at least 12 months of follow up). Participation was defined as a preterm infant obtaining at least one bottle of vitamin D during the first year of life. Secondary outcomes included adherence to the program (obtaining two or more prescription over the same time period) and whether participation or adherence varied by gestational age.

The insured vitamin preparations (400 IU/d of vitamin D) are all 50 dose bottles (1 ml = 1dose); the preparations included D-Vi-Sol®, Jamp-Vitamin D® and PediaVit D® and the multivitamin preparations Jamp-Vitamins A-D-C®, PediaVit® and Tri-Vi-Sol®. Baby Ddrops® (1drop = 400 IU) were added to the formulary in late 2009 but was not available during the study time frame.

Covariates for all outcomes include maternal age, post-secondary education, living status (couple vs. single), urban vs. rural, registrant vs. welfare recipient (RAMQ status), calendar year, specialty of prescribing physician, gestational age, birth weight, and sex of infant. Information on feeding method, parity and ethnicity were not available for use as potential confounders. The season of procurement of the first bottle of supplement was included to account for possible reliance on cutaneous production of vitamin D; October1 to March 31 was deemed the non-synthesizing period for vitamin D based on the low zenith angle of the sun for Quebec [14].

### Statistical methods

Descriptive statistics were used to compare the all preterms exposed to vitamin D supplements vs. those who were not. All analyses were repeated to determine if these varied by gestational age. Results are expressed as means and standard deviations or medians with range or percentages as appropriate. Multivariate logistic regression models were utilized to assess temporal trends in program participation and adherence and to identify additional predictors for both participation and adherence. Generalized estimating equations (GEE) were utilized to account for familial clustering (by mothers). Appropriate regression diagnostics were performed; statistical analyses were done using SAS (SAS institute Inc., Version 9.2, Cary, NC, USA).

### Ethics

The Research Ethics Committees at the Montreal Children’s Hospital, McGill University Health Center and CHU-Ste-Justine granted approval. The access to the data used to create the Quebec Pregnancy Cohort was authorized by the Commission d’Accès à l’Information and approved by the CHU-Ste-Justine ethics committee.

## Results

During the time frame of interest, 149307 infants were born in Quebec; 11504 (7.7%) were born at less than 37 weeks gestational age. After applying eligibility criteria, 10288 infants were available for analysis; the majority of these were born late preterm (n = 7819). Tables 1 and 2 present maternal, infant (Table 1) and physician characteristics (Table 2) regarding uptake of the free supplement program.

**Table 1:** **Characteristics of women and infants with respect to vitamin D exposure in infancy**

**Characteristics**	**Early preterm** ***n = 2409***			**Late preterm** ***n*** **= 7819**			**Total** ***n*** **= 10288**		
	**Exposed infants** ***n*** **= 902**	**Unexposed infants** ***n*** **= 1507**	***p*** **value**	**Exposed infants** ***n*** **= 1618**	**Unexposed infants** ***n*** **= 6201**	***p*** **value**	**Exposed infants** ***n*** **= 2520**	**Unexposed infants** ***n*** **= 7708**	***p*** **value**
Mothers’ characteristics									
Age, years, mean ± SD	27.7 ± 6.0	28.0 ± 6.1	0.284	28.1 ± 5.9	27.7 ± 5.7	0.010	28.0 ± 6.0	27.8 ± 5.8	0.136
Welfare recipient, % (*n*)	26.5 (239)	25.7 (387)	0.669	27.7 (448)	24.0 (1490)	0.002	27.3 (687)	24.4 (1877)	0.003
Mothers living alone, % (*n*)	22.1 (199)	21.3 (321)	0.665	19.1 (309)	20.2 (1251)	0.337	20.2 (508)	20.4 (1572)	0.802
Post-secondary education level, % (*n*)	36.1 (326)	35.8 (539)	0.851	40.8 (660)	35.4 (2193)	< 0.001	39.1 (986)	35.4 (2732)	< 0.001
Urban living, % (*n*)	76.9 (694)	80.5 (1213)	0.046	78.2 (1266)	78.5 (4866)	0.845	77.8 (1960)	78.9 (6079)	0.259
Infants’ characteristics									
Gestational age, weeks, mean ± SD	30.4 ± 2.5	30.6 ± 2.7	0.25	35.3 ± 0.8	35.4 ± 0.7	< 0.001	33.6 ± 2.8	34.5 ± 2.4	< 0.001
Weight at delivery, grams, mean ± SD	1577.9 ± 711.8	1657.7 ± 612.5	< 0.001	2573.4 ± 552.8	2649.1 ± 477.6	< 0.001	2217.0 ± 777.9	2455.2 ± 641.4	< 0.001
Male gender, % (*n*)	52.6 (474)	54.9 (828)	0.252	50.9 (823)	53.8 (3335)	0.044	51.5 (1297)	54.0 (4163)	0.037

**Table 2:** **Characteristics of physicians prescribing and adherence and participation**

**Characteristics**	**Early preterm** ***n = 2409***			**Late preterm** ***n*** **= 7819**			**Total** ***n*** **= 10288**		
	**Exposed infants** ***n*** **= 902**	**Unexposed infants** ***n*** **= 1507**	***p*** **value**	**Exposed infants** ***n*** **= 1618**	**Unexposed infants** ***n*** **= 6201**	***p*** **value**	**Exposed infants** ***n*** **= 2520**	**Unexposed infants** ***n*** **= 7708**	***p*** **value**
Specialty of the infants’ physician, % (*n*)									
General Practitioner	23.9 (216)	17.7 (267)	< 0.001	39.2 (635)	43.4 (2692)	< 0.001	33.8 (851)	38.4 (2959)	< 0.001
Paediatrician	73.5 (663)	53.8 (811)		57.5 (930)	47.7 (2958)		63.2 (1593)	48.9 (3769)	
Obstetrician- Gynaecologist	0.3 (3)	0.0 (0)		1.2 (20)	0.0 (0)		0.91 (23)	0.0 (0)	
Other specialist	2.2 (20)	28.5 (429)		2.0 (33)	8.9 (551)		2.1 (53)	12.7 (980)	
% of infants obtaining 2 or more bottles of Vitamin D (*n*)	61.6 (556)	-	-	49.7 (805)	-	-	54.0 (1361)	-	-
Among infants with 1 or more vitamin D prescription									
Age at the time of the first prescription. days. median (min-max)	62 (8–397)	-	-	35 (1–400)	-	-	49 (2–400)	-	
Number of vitamin D prescriptions	2 (1–12)	-	-	1 (1–15)	-	-	2 (1–15)	-	-
median (min-max)									
Infants initiating vitamin D during non-synthesizing period ……….% (*n*)*	51.3 (463)	-	-	52.4 (848)	-	-	52.0 (1311)	-	-

^*^Non synthesizing period: October 1^st^ to March 31^st^.

Roughly a quarter (24.5%) of all preterms participated; a higher proportion of early preterms obtained at least one bottle of vitamin D compared to those born after 34 weeks (37.4% vs. 20.7%, p < 0.0001) Additionally, a higher percentage of early preterm infants were likely to obtain more than 1 bottle of supplements via this program (61.6%) compared to late preterms (49.7%, p <0.0001). The median number of bottles procured was higher with 2 (range; 1–12) vs. 1 (range; 1–15) in those infants born earlier. The median time to procure the first supplement was longer in those born earlier (62 vs. 35 days). The only consistent predictors in both groups (assessed separately or together) for participation (obtaining 1 or more bottles over 12 mo) were specialty of infant’s physician and a lesser weight at delivery (Table 3).

**Table 3:** **Predictors of participation in the vitamin D program (infants with one or more prescription)**

**Characteristics**	**Early preterm**		**Late preterm**		**Total**	
	**OR**	**CI**	**OR**	**CI**	**OR**	**CI**
Maternal age (year)	1.00	0.98-1.01	1.01	1.00-1.02	1.01	1.00 - 1.02
Welfare recipient vs. adherent	0.84	0.67-1.05	1.12	0.97-1.29	1.05	0.93 – 1.19
Living alone vs. couple	1.10	0.89-1.37	0.93	0.80-1.09	0.99	0.87 – 1.12
Level of education: ≤12 years vs. >12 years	1.02	0.84-1.23	0.77	0.68-0.87	0.83	0.75 – 0.93
Rural vs. urban resident	1.22	0.97-1.53	1.04	0.90-1.19	1.08	0.96 – 1.22
Duration of pregnancy (week)	1.00	0.93-1.09	0.88	0.81-0.96	0.91	0.88 – 0.95
Birth weight (grams)	1.00	1.00-1.00	1.00	1.00-1.00	1.00	1.00 – 1.00
Male gender infants	1.02	0.86-1.21	0.95	0.85-1.06	0.97	0.88 – 1.07
Year of delivery	0.93	0.91-0.96	0.89	0.87-0.90	0.90	0.89 – 0.92
Non synthesizing* vs. synthesizing period	1.01	0.84-1.22	1.07	0.95-1.20	1.06	0.96 – 1.17
Infants followed up by General practitioner (reference)						
Paediatrician	0.92	0.74-1.16	1.23	1.09-1.39	1.21	1.09 - 1.35
Other specialist	0.08	0.05-0.12	0.41	0.31-0.55	0.20	0.16 - 0.27

*Non synthesizing period: October 1^st^ to March 31^st^.

The single consistent predictor for ‘total’, early or late premature infants was calendar year of delivery after adjustment; this was negatively associated (OR = 0.90 per year, 95% CI: 0.89-0.90). For late and ‘total’ preterms, higher maternal age, more education, prescription provided by a pediatrician or shorter duration of pregnancy were positive predictors for participation after adjustment for the other variables in the model. Adherence (obtaining 2 or more prescriptions over 12 months compared to those obtaining 1 prescription or no vitamin D) was also explored using a multivariate logistic regression (Table 4).

**Table 4:** **Predictors of adherence in the vitamin D program (infants obtaining 2 or more prescriptions)**

**Characteristics**	**Early preterm**		**Late preterm**		**Total**	
	**OR**	**CI**	**OR**	**CI**	**OR**	**CI**
Maternal age (year)	1.03	1.01-1.05	1.03	1.02-1.05	1.03	1.02 – 1.04
Welfare recipient vs. adherent	0.81	0.62-1.05	1.06	0.88-1.28	0.98	0.84 – 1.15
Living alone vs. in couple	1.01	0.76-1.33	0.91	0.74-1.12	0.96	0.82 – 1.13
Level of education: ≤12 years vs. >12 years	0.92	0.75-1.14	0.76	0.64-0.89	0.81	0.71 – 0.93
Rural vs. urban resident	1.03	0.79-1.36	1.08	0.90-1.31	1.07	0.92 – 1.24
Duration of pregnancy (week)	0.98	0.90-1.06	0.84	0.76-0.94	0.91	0.88 - 0.95
Birth weight (grams)	1.00	1.00-1.00	1.00	1.00-1.00	1.00	1.00 – 1.00
Male gender infants	1.11	0.90-1.36	1.04	0.90-1.21	1.04	0.92 – 1.17
Year of delivery	0.94	0.90-0.97	0.90	0.88-0.92	0.91	0.89 - 0.93
Non synthesizing* vs. synthesizing period	1.07	0.87-1.32	1.02	0.87-1.19	1.03	0.91 – 1.16
Infants followed up by General practitioner (reference)						
Paediatrician	1.08	0.84-1.39	1.29	1.09-1.52	1.27	1.11 - 1.45
Other specialist	0.11	0.06-0.19	0.47	0.32-0.69	0.23	0.16 - 0.32

*Non synthesizing period: October 1^st^ to March 31^st^.

For both gestational age categories and ‘total’ preterms, an older mother was positively associated, and the year of delivery was negatively associated. When evaluating adherence, the only positive predictors were a mother with more education, a shorter duration of pregnancy, and having a pediatrician care for the late and ‘total’ preterms.

The Figure 1 presents the number of infants obtaining 1 or more bottles; a steep drop was noted in the number of infants obtaining 2 or more bottles over a 12 mo. period.

## Discussion

Overall, preterm infants had low participation in the free vitamin D program, with only ~25% of them obtaining at least one bottle of supplement. Interestingly, nearly twice the number of early preterm (born <34 weeks) procured bottles via this program compared to late preterms (37.4% vs. 20.7%). As previously noted, only 18.2% of term infants from Quebec participated in this program over the same time frame [8]. Both late preterm and term infants also obtained a median number of 1 bottle (50 doses) of vitamin D during their 12 month follow up, while those born at <34 weeks procured enough (median) for 100 doses.

To date, there do not appear to be other manuscripts evaluating the programs promoting vitamin D supplements specifically in premature infants. Programs for all infants in the UK and in Turkey have met with variable success [7,15]; Turkey in particular has recently dramatically reduced its rate of rickets (6.8% to 0.1%) and improved the vitamin D status of its young children. Despite comprehensive campaigning, Birmingham has had a more moderate degree of success, with a tripling of uptake (5% to 17%) [15]. Those born 35 weeks or older in Quebec had a similar rate to Birmingham’s, but without their educational component [15]. One might speculate that promotional efforts like Birmingham’s might increase uptake several fold. Steps such as informing all physicians that vitamin D is free of charge to families and measures at nurseries, local pharmacies and physician’s offices are logical targets to advertise the program and reinforce the need for vitamin D.

This low utilization is at odds with the reports from the Canadian Community Health Survey and 2 other Canadian surveys, which typically addressed uptake of vitamin D in healthy term infants [16-18]. The difference may reflect biases in obtaining the data, such as recall bias (increasing vitamin D utilization when parents were surveyed) or that the 2 smaller surveys focused on urban populations. Our findings may also have excluded families purchasing vitamin D over-the-counter due to the failure of caregivers to provide prescriptions, beliefs that breast milk is complete, or challenges administering the available products.

Unfortunately, in all 3 groups (term, and 2 preterm groups), there was a significant decline in participation in the free program between 1998 and 2009 despite a province wide increase in exclusive breast feeding out to 6 mo (9 to 19% from 2003 to 2009) [8,9,16]. Older mothers, those with more education, and a prescription from a pediatrician increased the odds of both participation and adherence; there was a striking degree of consistency amongst predictors across all gestational ages, including term [7,8,19]. Because of the small numbers of early preterms, some of these predictors were only significant in the late preterm or total group.

The participation rate and higher numbers of bottles obtained in early preterms compared to late preterm or older infants might suggest possible explanations: Some NICUs or hospital nurseries may be promoting breastfeeding or vitamin D differently in these groups; parents and health-care givers may also perceive bone health risk differently in these vulnerable infants [20-23].

Specifically some hospitals have developed Family Care units with emphasis on breastfeeding promotion [23]. We hypothesize that these units may have placed particular emphasis on supplementation given that breast milk typically has low concentrations of vitamin D [23]. Additionally, evidence that breast milk may prevent disorders such as necrotizing enterocolitis or respiratory illnesses may also increase breastfeeding, particularly in those infants born at a younger gestational age [22]. Some literature suggests that late preterm infants are at times sent home relatively quickly with perhaps less support for breastfeeding, which may result in an earlier move to fortified formula [20,21]; this may then reduce the promotion of vitamin D for near-term infants. Unfortunately, we are not aware of the specific feeding modality in our populations.

Differential uptake may also reflect actual fractures or problems with mineral homeostasis during the hospitalization, prompting parents of early preterm infants to more eagerly adopt vitamin D supplements and continue them for a longer duration [1]. In general, parents of preterm infants perceive them to be more vulnerable than term infants, which may lead to more vigorous utilization of vitamin supplements [24]. Detailed data at an individual level were not available to us to support these hypotheses.

Our data were too limited to evaluate the impact of declining vitamin D uptake over time in just our preterm cohort; we previously reported the modest increase in rickets between 1998 and 2009 in infants of all gestational age in the QPC [8]. Additionally, we cannot comment in detail on the timing of procuring the first bottle of supplements. In term infants, there was a significant delay (median time was 36 d), which is similar to the median delay of 35 d in late preterm infants [8]. We do not know the age of discharge from hospital for the preterm infants, and the delay out to 66 d in the early preterms likely represents longer hospital stays.

Given the relatively better uptake in early preterm infants; we need to understand what prompted these families to obtain the supplements, unlike parents of later gestational infants. These observations and the previously described risk factors (younger mothers and those of lower SES) may allow Quebec and other jurisdiction to focus efforts to increase supplementation rates [7,8]. Moreover, because premature infants are at risk for fragility fractures out to 1-2y of life and there is still uncertainty about their optimal intake; 400 IU/d seems to be a reasonable target [25]. The utilization of special premature infant formula enriched in vitamin D and minerals post-discharge might replace the need for supplementation, but this, remains controversial [2].

There are some limitations with our data, such as not knowing the breastfeeding status of the infants, if the families received a prescription, if they actually administered the supplements once procured, or if they obtained vitamin D outside of the free program. The latter would still be perceived to be a failure of the program.

## Conclusion

All preterm infants had low uptake of the free supplement program, but early preterms demonstrated higher participation rates and better adherence. This would suggest that hospital-based interventions can improve vitamin D supplement uptake. Clearly, additional targeted reinforcement is warranted because preterm infants remain at risk for fragility fractures, and infants of young mothers with low education levels had lower levels of participation in the program [26]. Moreover, free vitamin D by prescription is clearly not sufficient without additional reinforcement; endorsement from a pediatrician appears to be particularly beneficial. These data help identify vulnerable infants and families that require additional support to promote adequate vitamin D intake.

## Abbreviations

D: Day
IU: International units
ICD: International classification diseases
L: Litre
Mo: Month
NHS: National Health Service
NICU: Neonatal intensive care unit
QPC: Quebec Pregnancy Cohort
RAMQ: Régie de l’Assurance Maladie du Québec
SES: Socioeconomic status
Y: Year
